# Psychotropic Polypharmacy in a 5-Year-Old Boy with a SHANK3 Variant and Complex Mental Health Problems

**DOI:** 10.3390/children13101297

**Published:** 2026-09-24

**Authors:** Wolfgang Briegel, Erdmute Kunstmann

**Affiliations:** 1 Department of Child and Adolescent Psychiatry, Psychosomatics and Psychotherapy, Leopoldina Hospital, 97422 Schweinfurt, Germany; 2 Department of Child and Adolescent Psychiatry, Psychosomatics and Psychotherapy, Julius-Maximilians-University, 97080 Würzburg, Germany; 3 Department of Psychology, University of Bielefeld, 33615 Bielefeld, Germany; 4 Practice for Human Genetics, 97080 Würzburg, Germany

**Keywords:** SHANK3, conduct disorder, attention deficit/hyperactivity disorder, psychotropic polypharmacy, child, case report

## Abstract

Background: Dysfunction of the SHANK3 gene has been linked to several neuropsychiatric and neurodevelopmental disorders, such as attention deficit/hyperactivity disorder (ADHD), autism spectrum disorder, and epilepsy. SHANK3 haploinsufficiency is the known etiology of Phelan–McDermid syndrome. Case presentation: This report presents a five-year-old boy in whom the c.2062G>C; p.Val688Leu variant was identified in a mosaic state in the SHANK3 gene (40% of heterozygous cells) and classified as a variant of uncertain significance (VUS). The patient was diagnosed with complex mental health problems, which, analogous to typical SHANK3 disruption, included ADHD, oppositional defiant disorder, developmental coordination disorder, Non-REM sleep arousal disorder, insomnia disorder, and avoidant/restrictive food intake disorder. Starting at the age of three years, his psychiatric treatment had included various medication trials over the course of two years. Ultimately, a substantial reduction in emotional outbursts, externalizing behaviors, and parental stress occurred following a multimodal treatment approach comprising psychotropic polypharmacy (PP), i.e., lisdexamfetamine, melatonin, and risperidone, along with behavioral therapy interventions, speech therapy, and occupational therapy. Under this PP, constipation was documented as the only adverse reaction. At the 15-month follow-up, improvements had been quite stable, despite risperidone medication being stopped about 19 weeks after initiation. Conclusions: As shown in this case report, even very young children with complex neuropsychiatric problems and SHANK3 variants might not be treated sufficiently by therapeutic interventions alone, but might require a combination of therapeutic and psychopharmacological interventions to achieve a substantial symptom reduction. However, PP must be approached with a high degree of caution and subjected to close monitoring in children with SHANK3 variants, who are susceptible to an elevated risk of adverse outcomes. Moreover, given the paucity of research in this population, it seems advisable to follow prevailing general guidelines in child and adolescent psychiatry.

## 1. Introduction

Mental disorders have been shown to exhibit high levels of comorbidity and genetic overlap (e.g., [1]), resulting in significant challenges to the effective diagnosis and treatment of patients. This complexity often necessitates the administration of psychotropic polypharmacy (PP), which is most commonly defined as the use of two or more psychotropic medications per day [2]. PP is also quite common in children and adolescents with mental health disorders [3,4,5], with studies indicating that pediatric PP (PPP) is associated with several factors, including male gender, older age, low socio-economic status, conduct disorders, and psychiatric comorbidity [3,5]. Rationales for using PPP include insufficient or absent treatment response, management of psychiatric comorbidities, and mitigation of adverse effects from concomitant medications [6]. However, there are several risks associated with PPP, including drug–drug interactions, issues with adherence, and a paucity of controlled studies supporting efficacy [4].

In addition to rare genetic variations, such as numeric and structural chromosomal aberrations and rare copy number variants, millions of common single-nucleotide polymorphisms (SNPs) also exist. Current knowledge on the functional effects of these variants is still limited, and about 80% of them have been interpreted as ‘variants of uncertain significance’ (VUS) [7,8]. However, a considerable number of SNPs have been identified through genome-wide association studies as associated with mental disorders [8]. In instances where feasible, psychiatrists have endeavored to formulate specific recommendations for the pharmacological treatment of mental disorders in individuals with identified genetic variations. However, without substantial scientific research, these recommendations have often been based on clinical practice. They may deviate considerably from established guidelines of child and adolescent psychiatry associations. This assertion applies to a variety of genetic disorders, including Phelan–McDermid syndrome [PMS; OMIM *606232] [9,10,11]. This condition is typically characterized by a heterozygous microdeletion of contiguous genes (including SH3 and Multiple Ankyrin Repeat Domains 3 gene (SHANK3; MIM *606230), or parts of this gene), located in the distal portion of the long arm of chromosome 22 [9,10]. Other cases of PMS result from single-nucleotide variants resulting in haploinsufficiency of the SHANK3 gene [9]. The dysfunction of SHANK3, a multi-domain synaptic scaffold protein that plays a critical role in maintaining the structural and functional integrity of synapses [12,13], is associated with a variety of neuropsychiatric and neurodevelopmental disorders, including autism spectrum disorder (ASD), attention deficit/hyperactivity disorder (ADHD), cognitive deficits, epilepsy, and sleep disorders [14,15]. However, if deleted together, other genes, such as MAPK8IP2 (OMIM *607755), may also influence neurodevelopment in PMS [9]. In view of the paucity of knowledge about the effectiveness of pharmacologic interventions in individuals with PMS and psychiatric disorders, especially in children and adolescents [11], specific recommendations for the treatment of the following symptoms have been formulated based on clinical consensus: attention deficit, hyperactivity, and impulsivity; irritability, aggression, and self-injury; anxiety; and catatonia [16]. In the context of ADHD, these consensus guidelines advocate for the utilization of alpha-2 adrenergic receptor agonists as first-line treatment. Conversely, the use of stimulant medication is recommended as a second-line intervention [16]. In the event of a partial response or non-response, a combination of alpha-2 adrenergic receptor agonists and stimulants is preferred to the application of viloxazine or atomoxetine (third choice) or antipsychotics as monotherapy or in combination with the aforementioned drugs (fourth choice) [16]. Regarding irritability, aggression, and self-injury, the prevailing recommendations for PMS primarily advocate for the use of alpha-2 adrenergic receptor agonists. In the event of a partial response or non-response, the recommended second-line drugs include benzodiazepines, baclofen, propranolol, CBD, N-acetylcysteine, and naltrexone. Mood stabilizers (third choice) and antipsychotics (alone or in combination with mood stabilizers; last-line intervention) are considered less effective [16]. To date, there have been no studies evaluating these recommendations for children and adolescents with PMS. Furthermore, there is a paucity of research on the effects of psychopharmacotherapy in children and adolescents with SHANK3 variants and complex mental disorders, rendering case studies a valuable source of information for this field.

## 2. Case

### 2.1. Patient Presentation

The patient was a five-year-old Caucasian male with average socioeconomic status and rural upbringing. He was referred for child psychiatric day treatment from an outpatient child psychiatric facility specializing in ASD. The parents reported that the boy had a pattern of recurrent, intense emotional outbursts in the home environment, including massive destructive behavior (e.g., demolition of doors) and severe physical aggression, which caused significant stress to the family. This behavior had previously also been observed in child-care settings. The parents articulated a need to refine the medication regimen and improve intra-familial interaction.

### 2.2. Personal History and Family Environment

The patient was the first of two children born to his parents, who were not related by blood. The parents were married and lived together. The mother had suffered from an eating disorder and depression in her youth. The father stated that as a child he had shown abnormalities in speech, early regulation, and social behavior.

The patient was born at 40 + 6 weeks’ gestation following a complication-free first pregnancy of his 29-year-old mother after in vitro fertilization. The vaginal delivery was spontaneous and uncomplicated, and the infant demonstrated good postnatal adaptation (APGAR score: 10/10/10). The newborn weighed 3020 g, was 50 cm in length, and had a head circumference of 33 cm. He was breastfed until age 3.5 years, as he consistently demonstrated a lack of interest in food and highly selective eating. He was particularly challenging to soothe and showed abnormalities in his sleep/wake rhythm, including night terrors. Moreover, language development exhibited abnormalities regarding expressive language. Over time, the following abnormalities became evident: impaired communication, a lack of interest in other children, and a tendency to become agitated when confronted with change. At the age of three years and 5 months, after a comprehensive assessment comprising both the Autism Diagnostic Observation Schedule-2 (ADOS-2; [17]) and the Autism Diagnostic Interview-Revised (ADI-R; [18]), the boy was diagnosed with ASD by a duly registered child and adolescent psychiatrist. Melatonin three mg daily was prescribed as a treatment for the patient’s sleep problems, which improved sleep patterns considerably. Additionally, the boy was referred to an outpatient clinic specializing in ASD, where he was primarily diagnosed with conduct disorder with childhood onset. Preliminary diagnoses of ADHD and ASD were also made. Genetic testing revealed a normal karyotype and, by exome sequencing, the variant c.2062G>C; p.Val688Leu in the SHANK3 gene in a mosaic state (40% of heterozygous cells). In accordance with established standards and guidelines for interpreting sequence variants [7], a geneticist classified this finding as a VUS, although it may be associated with the patient’s clinical features. Over the course of the following 22 months of treatment at the aforementioned outpatient clinic, the diagnosis of ADHD was confirmed, but not the ASD diagnosis. In accordance with consensus recommendations for PMS, but contrary to German child and adolescent psychiatry guidelines on ADHD and conduct disorder, a treatment regimen involving the administration of guanfacine as a first-line intervention was initiated [16,19,20]. However, as the patient exhibited a marked instance of constipation that did not improve with the administration of additional macrogol, the decision was made to discontinue the use of guanfacine and transition to the administration of methylphenidate. According to the parents’ reports, improvement was observed; however, it was time-limited. Consequently, methylphenidate administration was discontinued, and a trial with lisdexamfetamine was initiated. Under medication at 20 mg per day, the parents reported a high rate of desired outcomes for ADHD. However, they noted no significant change regarding conduct disorder symptoms.

### 2.3. Diagnostic Psychiatric Process at Admission

Physical examination revealed no abnormalities, and vaccination status was confirmed as complete. Cranial MRI, electroencephalograms (EEGs), including sleep deprivation EEGs, abdominal ultrasound, ECG, and ENT examination revealed no abnormalities. However, a mental examination revealed primarily significant expansive behavior problems (conduct problems, attention problems, significant impulsivity and hyperactivity), sleep problems, coordination problems, and highly selective eating, but no autism spectrum disorder. A comprehensive psychological evaluation, including the Eyberg Child Behavior Inventory ([21]; both parents: Intensity score: *t* = 95, Problem score: *t* = 78), revealed significant externalizing behavior problems, normal intelligence, but significant coordination problems. A multidisciplinary consensus process led by a board-certified child and adolescent psychiatrist assigned the following diagnoses in accordance with the Diagnostic and Statistical Manual of Mental Disorders, 5th edition: conduct disorder with childhood onset; ADHD, combined type; developmental coordination disorder; Non-REM sleep arousal disorder; insomnia disorder; avoidant/restrictive food intake disorder [22].

### 2.4. Treatment Process and Outcome

During the 16-week day treatment, the treatment plan encompassed a range of behavioral therapy interventions, targeting both individual and family levels, as well as speech therapy and occupational therapy. Melatonin 3 mg daily and lisdexamfetamine 20 mg per day were continued because a discontinuation test of lisdexamfetamine suggested a significant effect. Seven weeks after admission, with the interventions implemented thus far appearing to be insufficient for a clinically significant reduction in aggressive behaviors toward parents and other children, following the receipt of consent, risperidone, an off-label medication, was initiated at 0.25 mg in the evening with subsequent escalation to 0.5 mg after one week. The boy showed substantial, rapid clinical improvement in behavior and sleep at home and at the day treatment center with 0.5 mg of risperidone within seven days, and this improvement remained stable until discharge. Except for significant constipation, which was successfully mitigated by macrogol administration, no adverse reactions were documented. For a detailed timeline of psychopharmacological interventions, see Table 1. A parent-report measures-based psychological evaluation conducted before discharge revealed child behaviors to be within normal limits and parental stress to be average. To determine whether changes in child disruptive behavior (ECBI Intensity score) and parental distress (ECBI Problem score) exceeded the margin of measurement error, we calculated reliable change indices (RCI [23]). Because no German test–retest reliability results are available, we used Dutch results [24] to calculate RCIs, with patients with an RCI ≥ |1.96| considered recovered. To determine whether changes were clinically significant, the Jacobson, Roberts, Berns, and McGlinchey criteria [25] were applied, which imply that the child’s score is in the clinically significant range at the time-one assessment and in the normal range at the time-two assessment, and that the change in scores from time one to time two is statistically reliable as defined using the RCI. For more detailed information, see Table 2.

After fifteen months, the parents agreed to discuss the boy’s progress since his discharge. It was reported that despite the persistence of mild symptoms of conduct disorder in the domestic environment, the boy had succeeded in his transition to regular schooling and graduated from his initial school year. The teacher had provided positive feedback, and the patient had a close companion in his class with whom he was also acquainted during extracurricular activities. The parents continued lisdexamfetamine and melatonin, but discontinued risperidone about 10 weeks after discharge because they did not see any further need. This discontinuation did not cause any subsequent deterioration.

## 3. Discussion

With a special focus on PP, this case report describes the treatment course of a 5-year-old male receiving a combination of therapeutic (behavior therapy and developmental therapies) and psychopharmacological interventions. The patient presented with a VUS in the SHANK3 gene and a multifaceted psychiatric condition comprising the following DSM-5 disorders: conduct disorder with childhood onset; ADHD, combined type; developmental coordination disorder; Non-REM sleep arousal disorder; insomnia disorder; avoidant/restrictive food intake disorder. Although the clinical significance of the patient’s genetic variant remains indeterminate, the clinical presentation, together with the observation that missense point mutations in the proximity of the patient’s genetic locus are associated with conformational fluctuations and protein turnover at synapses, might support the hypothesis that SHANK3 dysfunction may have contributed to the clinical presentation [29].

The administration of psychopharmacotherapy was initiated due to the presence of sleep disturbances at the age of three. Subsequently, the patient was also prescribed medications for ADHD, including guanfacine, methylphenidate, and lisdexamfetamine. In contrast to guanfacine and methylphenidate, a significant reduction in ADHD symptoms followed the administration of lisdexamfetamine, without any undesirable side effects. However, persistent emotional outbursts, aggression, and irritability despite behavior therapy interventions necessitated the concomitant administration of risperidone at a low dose. Ultimately, this multimodal approach was followed by a substantial decrease in emotional outbursts and externalizing behaviors, with parents reporting the child’s behavior and parental stress as within the normal range. Moreover, as indicated by the ECBI, externalizing behaviors and parental stress showed a clinically significant change. Furthermore, after the administration of risperidone, a reduction in preexisting sleep problems (under medication with melatonin) was reported by the patient’s parents. However, the patient developed clinically significant constipation, which had to be treated with macrogol. Constipation is well known as a potential adverse effect of risperidone; however, it is also a common problem in patients with a deletion of SHANK3 [30].

So far, psychopharmacological recommendations specifically targeting psychiatric disorders in combination with SHANK3 variants are lacking. However, expert consensus recommendations have been established for the management of various psychiatric symptoms in patients with PMS [16], which is typically, but not exclusively, associated with SHANK3 dysfunction [9]. The decision to administer the atypical antipsychotic risperidone is in contrast to the prevailing recommendations for PMS, which consider risperidone to be a fourth-choice drug for irritability, aggression, and self-injury [16]. The following aspects were considered in arriving at this decision: Risperidone has received approval in Germany for the “short-term treatment” (up to 6 weeks) of persistent aggression in children with conduct disorder and sub-average intellectual functioning, with the age of the patient being a minimum of five years. However, in clinical practice, risperidone is frequently prescribed for long-term treatment and for young patients with average or above-average intelligence who exhibit persistent aggressive behavior. Secondly, the German child and adolescent psychiatry guidelines indicate that risperidone is regarded as the first-line medication for conduct disorders, even when used as an off-label medication, as in this particular case, in which the patient showed normal intelligence [9]. Finally, because the boy described in this case report did not meet criteria for PMS and showed only a VUS in the SHANK3 gene, PMS-specific guidelines did not seem more appropriate than general child and adolescent psychiatry guidelines.

To the best of the authors’ knowledge, there has been no report on the use of risperidone in a child or adolescent with a SHANK3 variant to date, whether associated with PMS or not. However, the findings of this study are consistent with those of a case report of an 18-year-old woman with PMS who exhibited significant improvements in mood and behavior after being administered 1 mg of risperidone daily [31]. The apparent positive effects of risperidone may be attributable to D2 receptor antagonism, thereby promoting NMDA transmission and reversing the glutamatergic dysregulation resulting from SHANK3 dysfunction, but this might be specific to low-dose risperidone administration, as animal experiments suggest [31,32]. Interestingly, in this case, risperidone medication was discontinued by the parents about 19 weeks after initiation without any deterioration of symptoms. Although the reason(s) for this finding remain unclear, possible explanations include that risperidone functioned as a booster by reducing parental stress and enabling parents to implement better behavior interventions, as well as maturation-related changes in the boy’s behavior.

When interpreting the findings, it is imperative to acknowledge the limitations inherent in this case report. First, it is crucial to acknowledge the methodological limitations intrinsic to case study designs. These include the absence of a control group and limited generalizability. Second, the pre- and post-treatment assessment instruments were identical only for the ECBI, and no follow-up assessment was possible. Third, in accordance with prevailing guidelines, the boy received not only PP but also behavioral therapy interventions targeting both the individual and family levels, as well as developmental therapies. While this is considered good clinical practice, it complicates clear differentiation between the effects of single interventions, such as risperidone treatment and behavioral therapy. However, after the initiation of risperidone treatment, child behaviors and parental stress were in the normal range as determined by parental assessments, thus justifying PP continuation. The decision was also made based on the fact that constipation was the only adverse drug reaction, which was quite easily manageable.

In light of the well-documented risks associated with PPP, including but not limited to drug–drug interactions and issues with adherence, PP must be approached with a high degree of caution and subjected to close monitoring. This seems to be particularly salient in cases involving children with SHANK3 variants, who are susceptible to an elevated risk of adverse outcomes. Furthermore, if specific SHANK3 variants of unknown significance have been identified, it seems reasonable to primarily adhere to prevailing general guidelines in child and adolescent psychiatry, rather than to those for PMS.

Given the dearth of extant knowledge concerning the effects of psychopharmacotherapy in children and adolescents with SHANK3 variants and complex mental disorders, this case study constitutes a significant contribution to the field by providing a rare clinical observation. Given the multimodality of treatment, the apparent effects of PP, particularly risperidone, should be considered cautiously and without causal overinterpretation. Further research with larger samples is necessary to comprehensively assess the efficacy, benefits, and adverse effects of psychopharmacological interventions and PP in this unique patient population.

## Figures and Tables

**Table 1:** Timeline of psychopharmacological interventions.

Drug	Reason for Initiation	Age at Initiation (Years; Months)	Dose (mg/day)	Clinical Improvement	Adverse Effects	Duration (Months)	Reason(s) for Discontinuation
Melatonin	Sleep–wake disorder/insomnia disorder	3; 7	1.0–3.0	Good	None	45	n. a.
Guanfacine	ADHD and conduct disorder	5; 5	1.0–2.0	Moderate	Constipation	2	Refractory massive constipation
Methylphenidate	ADHD and conduct disorder	5; 7	2.5–15.0	Moderate	None	2	Time-limited improvement
Lisdexamfetamine	ADHD and conduct disorder	5; 9	10.0–20.0	Good	None	19	n. a.
Risperidone	Conduct disorder	6; 0	0.25–0.5	Good	Constipation	Almost 5	Parents did not see any further necessity

Legend: Clinical Improvement: None = 0–10%, Marginal: 11–25%, Moderate: 26–50%, Good: 51–75%, Excellent: 76–90%; Absolute: 91–100%; ADHD = Attention Deficit/Hyperactivity Disorder; n. a. = not applicable.

**Table 2:** Results of psychological assessment before discharge.

Measure [Unit]	Results	Reliable Change Index [23]; Clinically Significant Change [25]
*Child behavior aspects:*		
Eyberg Child Behavior Inventory (ECBI) [21] [*t*-score]	Mother: Intensity score: 54, Problem score: 52 Father: Intensity score: 59, Problem score: 57	Mother: Intensity score: 8.04; Problem Score: 4.94; Clinically Significant Change Father: Intensity score: 7.12; Problem score: 4.10; Clinically Significant Change
FBB-ADHS-V [26] [stanine value]	Mother: Total: 3; Attention deficit: 4; Hyperactivity/Impulsivity: 3 Father: Total: 3; Attention deficit: 4; Hyperactivity/Impulsivity: 3	n. a. n. a.
Child Relationship Behavior Inventory [27] (CRBI) [*t*-score]	Mother: CRBI ratio: 46; Father: CRBI ratio: 55	n. a.
*Parent aspects:*		
Parenting Stress Questionnaire [28] [stanine value]	Mother: Parent stress: 5; Father: Parent stress: 5	n. a.

Legend: n. a. = not applicable.

## Data Availability

The data presented in this study are available on request from the corresponding author due to privacy and legal restrictions.
